# The value of G-CSF in women experienced at least one implantation failure: a systematic review and meta-analysis

**DOI:** 10.3389/fendo.2024.1370114

**Published:** 2024-04-16

**Authors:** Qing Su, Zhuo Pan, Rong Yin, Xuemei Li

**Affiliations:** ^1^ Chongqing University Central Hospital, Chongqing Emergency Medical Center, Chongqing, China; ^2^ Center for Reproductive Medicine, Chongqing Key Laboratory of Human Embryo Engineering, Chongqing Reproduction Genetics Institute, Chongqing Health Center for Women and Children, Women and Children's Hospital of Chongqing Medical University, Chongqing, China

**Keywords:** granulocyte colony-stimulating factor, implantation failure, *in vitro* fertilization, intracytoplasmic sperm injection, pregnancy outcome

## Abstract

**Objective:**

Despite the developments of *in vitro* fertilization (IVF) protocols, implantation failure remains a challenging problem, owing to the unbalance between the embryo, endometrium, and immune system interactions. Effective treatments are urgently required to improve successful implantation. Recently, many researchers have focused on granulocyte colony-stimulating factor (G-CSF) to regulate immune response and embryo-endometrium cross-talk. However, previous studies have reported inconsistent findings on the efficacy of G-CSF therapy on implantation failure. The objective of this review was to further explore the effects of G-CSF according to administration dosage and timing among women who experienced at least one implantation failure.

**Methods:**

We systematically searched MEDLINE, Embase, the Cochrane Central Register of Controlled Trials, Scopus, and Web of Science for randomized controlled trials of G-CSF on implantation failure up to July 21, 2023. Odds ratios (ORs) and 95% confidence intervals (CIs) were calculated and the heterogeneity of the studies with the I^2^ index was analyzed.

**Results:**

We identified a total of 2031 studies and finally included 10 studies in the systematic review and meta-analysis. G-CSF administration improved the clinical pregnancy rate (CPR), implantation rate (IR), biochemical pregnancy rate (BPR), and live birth rate (LBR) in women with at least one implantation failure. Subgroup analyses showed that G-CSF treatment could exert good advantages in improving CPR [OR=2.49, 95%CI (1.56, 3.98), I^2^ = 0%], IR [OR=2.82, 95%CI (1.29, 6.15)], BPR [OR=3.30, 95%CI (1.42, 7.67)] and LBR [OR=3.16, 95%CI (1.61, 6.22), I^2^ = 0%] compared with the blank control group. However, compared with placebo controls, G-CSF showed beneficial effects on CPR [OR=1.71, 95%CI (1.04, 2.84), I^2^ = 38%] and IR [OR=2.01, 95%CI (1.29, 3.15), I^2^ = 24%], but not on LBR. In addition, >150μg of G-CSF treatment increased CPR [OR=2.22, 95%CI (1.47, 3.35), I^2^ = 0%], IR [OR=2.67, 95%CI (1.47, 4.82), I^2^ = 0%] and BPR [OR=2.02, 95%CI (1.17, 3.47), I^2^ = 22%], while ≤150μg of G-CSF treatment improved miscarriage rate (MR) [OR=0.14, 95%CI (0.05, 0.38), I^2^ = 0%] and LBR [OR=2.65, 95%CI (1.56, 4.51), I^2^ = 0%]. Moreover, G-CSF administration on the day of embryo transfer (ET) could increase CPR [OR=2.81, 95%CI (1.37, 5.75), I^2^ = 0%], but not on the day of ovum pick-up (OPU) or human chorionic gonadotropin (HCG) injection.

**Conclusion:**

G-CSF has a beneficial effect on pregnancy outcomes to some extent among women who experienced at least one implantation failure, and the administration dosage and timing influence the effect size.

**Systematic review registration**

https://www.crd.york.ac.uk/prospero/, identifier CRD42023447046.

## Highlights

- **Question/Objective**: What are the effects of different administration dosages and timing of granulocyte colony-stimulating factor (G-CSF) for women experienced at least one implantation failure?
- **Findings**: Ten RCTs evaluating the effect of different administration dosages and timing of G-CSF for women with at least one implantation failure were included. The present study demonstrated that G-CSF improved pregnancy outcomes. More specifically, >150μg of G-CSF treatment increased clinical pregnancy rate (CPR), implantation rate (IR), and biochemical pregnancy rate (BPR), while ≤150μg of G-CSF treatment improved miscarriage rate (MR) and live birth rate (LBR). In addition, G-CSF administration on the day of embryo transfer (ET) could increase CPR, but not on the day of ovum pick-up (OPU) or human chorionic gonadotropin (HCG) injection.
- **Meaning**: Based on the meta-analysis, G-CSF could improve pregnancy outcomes, and the administration dosage and timing influence the effect size.

## Introduction

Embryo implantation is a key process in reproduction, most of the pregnancy failures happen during the embryo implantation period (1). The incidence of implantation failures varies from 8 to 33% in the general population (2). Despite the developments of *in vitro* fertilization (IVF) protocols, the embryo implantation rate is only 20-30%, and only 40% even if the blastocyst is transferred. Around 10-15% of patients undergoing assisted reproductive technology (ART) procedures experience unexplained recurrent implantation failures (3–5). Given the challenges encountered with the incidence of implantation failure, additional efforts are urgently required to increase successful fertilization and implantation.

Embryo implantation is regulated by several factors, the balance between the embryo, endometrium, and immune system interactions are essential for successful implantation (2, 6). It is estimated that embryos account for one-third of implantation failures, while suboptimal endometrial receptivity and altered embryo-endometrial cross-talk are responsible for the remaining two-thirds (7–9). However, there are some other influential factors, such as anatomic structure, autoimmune factors, thrombophilic conditions and lifestyle (10), which means that a multidisciplinary approach is required for the management of implantation failure. Various interventions have been developed to improve implantation, especially for those with repeated implantation failure. These approaches include endometrial scratch injury (11), improving endometrial thickness in women with thin endometrium (12), intrauterine human chorionic gonadotrophin (13), intravenous Atosiban (14), preimplantation genetic screening (15), and the use of immunomodulators (16). Despite these therapeutic alternatives, implantation failure remains a challenging problem.

Granulocyte colony-stimulating factor (G-CSF) is a hematopoietic-specific cytokine synthesized by bone marrow cells, stromal cells, fibroblasts, and macrophages (17), and has been proven to originate from some reproductive tissue cells (18). Particularly, some pieces of evidence have shown that G-CSF and its receptor are located in luteinized granulose cells, trophoblastic cells and oocytes (19–21), indicating the importance of this cytokine in implantation. Moldenhauer LM et al. found that G-CSF temporarily suppresses the immune response and plays an essential part in embryo-endometrium cross-talk through its effects on type two T helper cells and endometrial angiogenesis (22). In 2009, G-CSF was first successfully used in patients with recurrent abortions (23), and later several clinical trials suggested that G-CSF administration may improve the success of IVF in thin endometrium (24, 25). The therapeutic effect of G-CSF in patients with recurrent implantation failure (RIF) has been investigated as early as 2000, and the results show that systematic administration of G-CSF can enhance the implantation rate dramatically (26). Since then, bulks of studies with controversial results have evaluated the effect of G-CSF on implantation failures due to poor endometrial thickness or other reasons. In the study of Aleyasin and coworkers, administration of single-dose subcutaneous G-CSF before implantation significantly increases implantation and pregnancy rates in infertile women with repeated IVF failure (27). However, Kalem and colleagues showed that the administration of G-CSF into the uterine cavity in RIF patients with normal endometrium did not alter the endometrial thickness, clinical pregnancy rate, or live birth rate (28).

Synthesized evidence is needed to help clinicians choose an appropriate treatment for infertility women with implantation failure. However, the current meta-analysis shows many shortcomings. For example, Kamath MS et al. showed that the pregnancy rate of RIF in the G-CSF group was significantly higher than that in the placebo group (29). Nevertheless, only two articles were included, which contributed to bias in the outcome. Recently, Hou, et al. indicated that G-CSF improved the clinical pregnancy rate (CPR) in patients with unexplained RIF for both the fresh and frozen embryo transfer cycles, and for both subcutaneous injection and intrauterine infusion (30). However, it failed to compare the effects of different dosages of G-CSF on pregnancy outcomes. Furthermore, the best administration timing for G-CSF remains uncertain. In this systematic review and meta-analysis, in addition to the CPR, biochemical pregnancy rate (BPR), implantation rate (IR), miscarriage rate (MR) and live birth rate (LBR), subgroup analysis was conducted according to administration dosage of G-CSF and timing of intervention to obtain further information on the influence of G-CSF on implantation failure patients.

## Methods

This systematic review adheres to the Preferred Reporting Items for Systematic Reviews and Meta-Analyses (PRISMA) and was registered with the International Prospective Register of Systematic Reviews (PROSPERO), number CRD42023447046.

### Search strategy and eligibility criteria

We selected relevant studies from the following databases: MEDLINE, Embase, the Cochrane Central Register of Controlled Trials, Scopus and Web of Science. We developed a search strategy from text and MeSH terms related to “granulocyte colony-stimulating factor,” “G-CSF,” “implantation failure,” “endometrium,” “*in vitro* fertilization,” “IVF,” and “intracytoplasmic sperm injection” up to July 21, 2023. Additionally, references and citations of the relevant literature retrieved were carefully searched to find more additional eligible literature.

We included studies that compared G-CSF to no intervention, placebo, or any other treatments. We identified eligible studies according to the following criteria: participants had to be subfertile women who experienced one or more implantation failures and have no uterine adhesions; the outcomes were medically confirmed pregnancy outcomes including CPR, IR, BPR, LBR, and MR; study design must be double-blind RCTs. Exclusion criteria were as follows: observation studies, animal studies, case reports, self-pro-post studies, conference abstracts and review articles; studies not published in English. For the overlapped sample sources, we included the report with more information and larger sample sizes.

Two independent investigators reviewed study titles and abstracts, and studies that satisfied the inclusion criteria were retrieved for full-text assessment. The agreement of both investigators determined final eligibility. Disagreements were referred to a third reviewer to reach consensus.

### Data extraction and assessment of risk bias

Two reviewers independently extracted the following data from each included study using a specifically designed form: study design, sample sizes, participant characteristics, the number of previous implantation failures, intervention details, and outcome parameters. Extracted data were abstracted directly onto previously designed standardized electronic form.

Two independent reviewers assessed the included studies for methodological quality using the Cochrane risk of bias tool, containing seven specific domains. We resolved any disagreements by consensus or by discussion with a third author. Each domain was assigned a judgment relating to the risk of bias for that study classified as low, high, or unclear risk. Disagreements were referred to a third reviewer to reach a consensus.

### Statistical analysis

The odd ratio(OR) and the corresponding 95% CI were calculated for all dichotomous data. The pooled ORs were calculated through a Mantel-Hansel fixed-effects model if there was no heterogeneity; otherwise, a random-effects model was adopted. We assessed statistical heterogeneity using the I^2^ statistic, with values greater than 50% regarded as moderate-to-high heterogeneity. We performed prespecified subgroup analyses according to the following parameters: dosage of administration of G-CSF and intervention timing. We did Egger tests to assess funnel plot asymmetry and defined significant publication bias with a p-value lowing 0.1. We conducted the statistical analyses with Review Manager(Revman5.3.3, Cochrane Collaboration, Copenhagen, Denmark, 2014) and Stata software (version 14.0, Stata Corp LP, Texas, USA, 1985-2015).

## Results

### Search results

**Figure 1:**
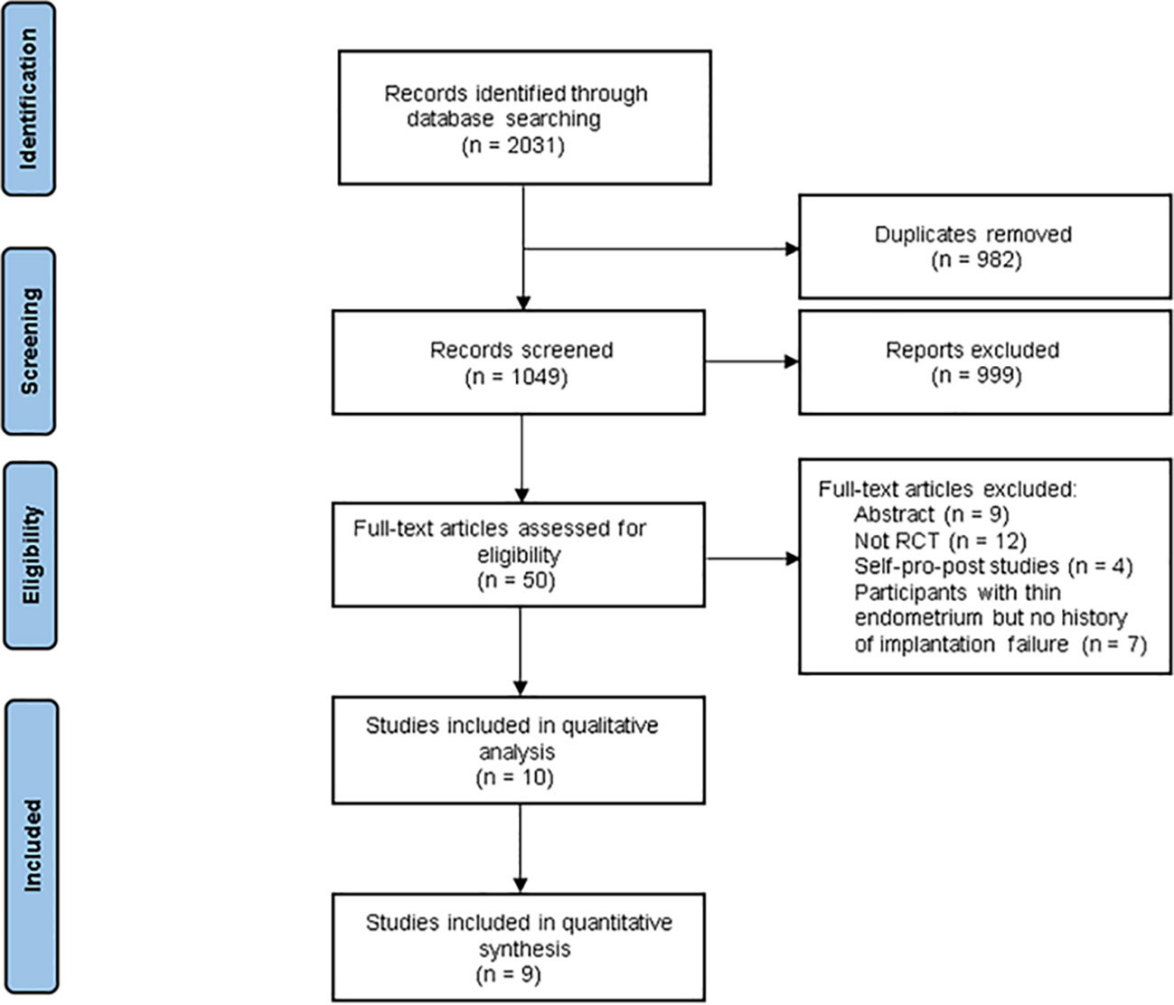
Flow diagram for study selection process.

In total, we identified 2031 studies, and 1049 studies remained after we removed duplicates. Subsequently, we excluded 999 studies after reviewing the titles and abstracts. Of the remaining 50 articles assessed for eligibility, 40 studies were further excluded after reading the full text for the following reasons: 9 were abstracts; 15 studies were not part of the literature about implantation failures; 12 studies were not RCTs, 4 of them were self-pro-post control studies. Finally, ten studies reported sufficient data (27, 28, 31–38), while nine studies were included in the quantitative synthesis (27, 28, 31–37). A flow diagram depicting the search and selection process is shown in **Figure 1**.

### Description of included studies

The meta-analysis contained 942 participants: 454 women in the intervention group,99 of whom were in the HCG group, and 389 were in the control group. Mean age ranged from 31.75 ± 5.16 to 35.5 ± 4.32 years old. Seven studies compared the G-CSF group with the control group (no intervention or placebo) (27, 28, 31, 33–36), one compared the G-CSF group with the HCG group (32), and one study included three arms: G-CSF group, placebo group and HCG group (37). In two studies, G-CSF was administered by subcutaneous injection (27, 31), while in all the other studies included, G-CSF was used by intrauterine perfusion. Seven studies analyzed fresh cycles (27, 28, 31, 32, 34, 36, 37), one compared frozen cycles (35), and one included both fresh and frozen cycles (33). Participants in all of these studies had endometrial thicknesses greater than 7mm (**Table 1**).

**Table 1:** Characteristics of included trials.

Author/Year	Studydesign	Sample sizes	ET cycle	Inclusion criteria	Intervention group	Control group	Outcomes
Aleyasin 2016 (27)	RCT	112	Fresh cycle	failure of implantation in at least three consecutive IVF attempts, in which three embryos of high-grade quality are transferred in each cycle	a single dose of 300µg G-CSF was administered subcutaneously one hour before the ET.	did not receive any additional treatment before the ET.	CPR, IR, BPR, EPR
Arefi 2018 (31)	RCT	52	Fresh cycle	more than two previous IVF/ICSI-ET failures despite transfer of at least two good-quality embryos in each attempt	a single dose of 300µg G-CSF was administered subcutaneously 30 min before embryo transfer	did not receive any additional treatment before the embryo transfer.	CPR, MR, LBR
Bakry 2022 (32)	RCT	100	Fresh cycle	RIF: clinical pregnancy failure after three cycles of IVF	uterine infusion of 100µg G-CSF on embryo transfer day	injected with 500 IU of intrauterine HCG on embryo transfer day	CPR, IR, BPR
Davari 2016 (33)	RCT	100	Fresh cycle (n=91) Frozen cycle (n=9)	RIF: three times implantation failure despite transfer of at least four good quality embryos	uterine infusion of 300µg G-CSF at the day of oocyte retrieval or starting progesterone	injected with normal saline; a catheter pass through the cervix without any injection	CPR, IR, BPR, MR
Eftekhar 2016 (34)	RCT	90	Fresh cycle	with history of at least two implantation failures	uterine infusion of 300μg recombinant human G-CSF at the day of oocyte retrieval	did not receive any additional treatment	EMT, CPR, IR
Huang 2020 (35)	RCT	163	Frozen cycle	≥two failed implantations (each time containing at least one high-quality embryo)	uterine infusion of 150µg G-CSF three days before the ET	uterine infusion of normal saline three days before the ET	EMT, CPR, IR, MR, LBR
Kalem 2020 (28)	RCT	157	Fresh cycle	RIF: failure to achieve a clinical pregnancy after the transfer of at least four good-quality embryos in a minimum of three fresh or frozen cycles	uterine infusion of 300µg G-CSF once before HCG injection	uterine infusion of normal saline once before HCG injection	EMT, CPR, BPR, MR, LBR, early premature birth rate
Karimi 2020 (36)	RCT	93	Fresh cycle	unexplained RIF : at least two pervious unsuccessful IVF/ICSI cycles,	uterine infusion of 150µg G-CSF just after ovarian puncture	uterine infusion of normal saline just after ovarian puncture	CPR, LBR, MR, OPR
Torky 2022 (37)	RCT	147	Fresh cycle	RIF: three or more failed attempts with at least four good quality embryos transferred	uterine infusion of 100µg G-CSF after oocyte retrieval	uterine infusion of 500IU HCG after oocyte retrieval; uterine infusion of normal saline after oocyte retrieval	CPR, IR, BPR, MR
Rezaei 2020 (38)	RCT	34	Frozen cycle	at least two failed IVF cycles with a minimum of three suitable embryos for transfer	uterine infusion of 300µg G-CSF and normal saline was injected subcutaneously	300µg G-CSF was injected subcutaneously and uterine infusion of normal saline	EMT, CPR, BPR

RCT: Randomized controlled trials; G-CSF: granulocyte colony-stimulating factor; HCG: human chorionic gonadotropin; IVF: in vitro fertilization;

ICSI: intracytoplasmic sperm injection; ET: embryo transfer; RIF: recurrent implantation failure; EMT: endometrial thickness; CPR: clinical pregnancy rate;

IR: implantation rate; BPR; biochemical pregnancy rate; MR: miscarriage rate; LBR: live birth rate; OPR: ongoing pregnancy rate; EPR: ectopic pregnancy rate.

One RCT could not be included in the quantitative analysis due to the absence of outcomes in the placebo group. Rezaei Z. et al. observed the beneficial effects of CPR in both the intrauterine group and the subcutaneous group, with no significant difference between the two groups. Besides, they also reported significantly lower drug side effects in the intrauterine group (38).

### Risk of bias in included studies

**Figure 2:**
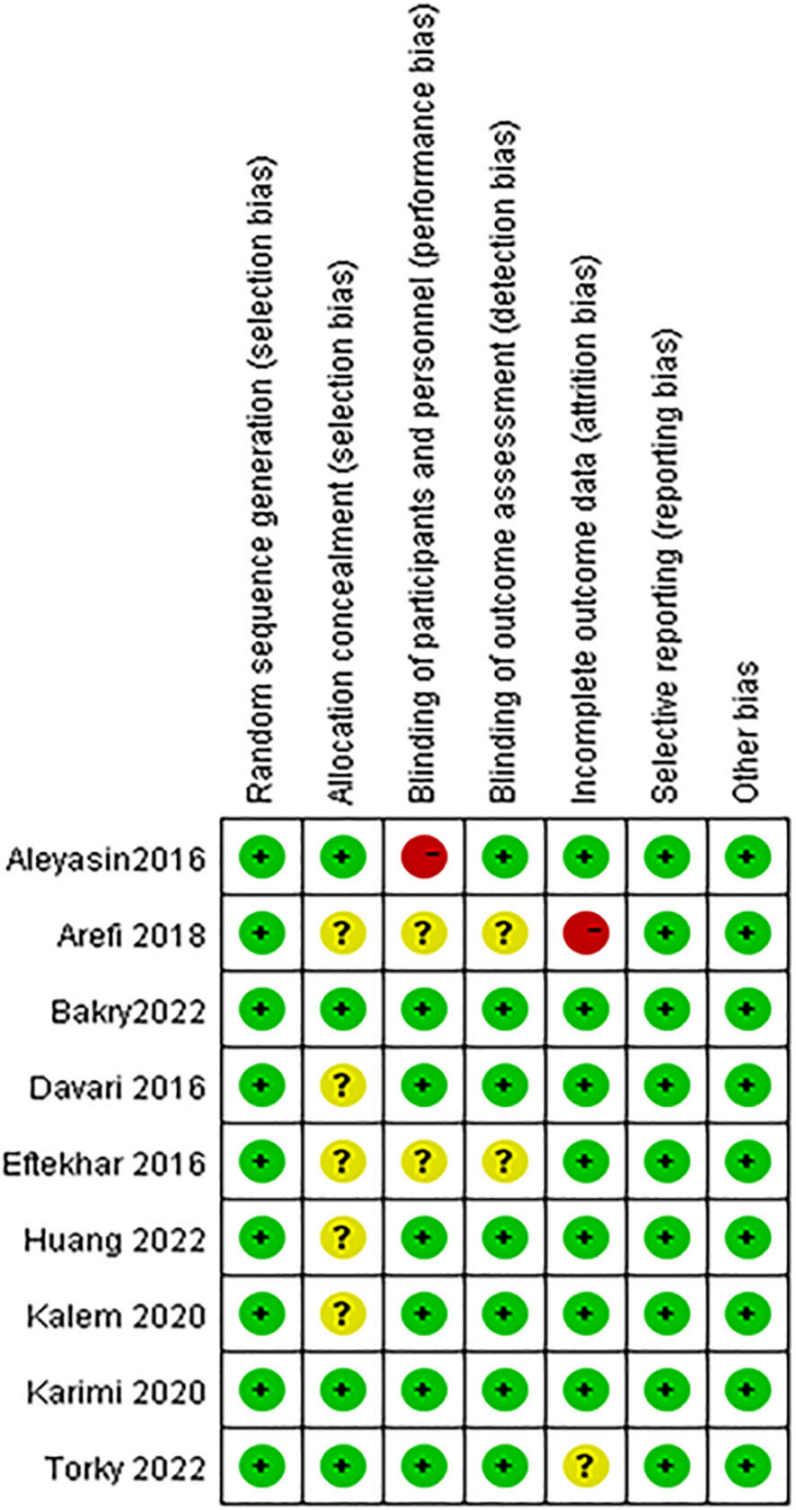
Risk of bias summary: review authors' judgements about each risk of bias item for each included study.

We assessed the risk of bias in all included studies, as demonstrated in **Figure 2**. All of the included studies reported adequate methods for random sequence generation. Five studies did not specify whether data collectors and outcome assessors were masked to treatment allocation (28, 31, 33–35). Two studies were rated at low risk of bias (32, 36), and two were judged to be at high risk because of open-label or incomplete outcome data (27, 31).

### G-CSF versus no intervention

#### CPR

**Figure 3:**
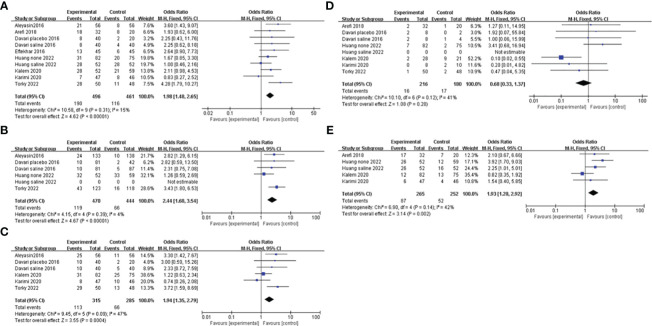
Forest plot showing individual and combined effect size estimates and 95% confidence intervals (CIs) in studies that evaluated the effect of G-CSF on pregnancy outcomes in women who experienced at least one implantation failure. **(A)** clinical pregnancy rate. **(B)** implantation rate. **(C)** biochemical pregnancy rate. **(D)** miscarriage rate. **(E)** live birth rate.

All of the studies, comparing the G-CSF group with the control group, described CPR. Considering the low heterogeneity (I^2 ^= 15%), a fixed-effects model was utilized for the meta-analysis, and the result showed that G-CSF could better improve the CPR [OR=1.98, 95%CI (1.48, 2.65)], as shown in **Figure 3A**.

#### IR

Four studies reported the IR and included 914 patients, 470 of whom were in the study group and 444 of whom were in the control group. A fixed-effects model was used for data synthesis, and the results indicated that G-CSF treatment improved the IR in patients with a history of implantation failure [OR=2.44, 95%CI (1.68, 3.54), I^2 ^= 4%]; see **Figure 3B** for details.

#### BPR

Five studies compared BPR after different interventions in two groups. A fixed-effects model was adopted for data analysis, and the results showed that BPR was increased by G-CSF administration [OR=1.94, 95%CI (1.35, 2.79), I^2 ^= 47%]; see **Figure 3C** for details.

#### MR

A total of six studies were reported and included 386 patients. A fixed-effects model was used for meta-analysis, and the results revealed that there was no significant difference in MR in the two groups with or without G-CSF [OR=0.68, 95%CI (0.33, 1.37), I^2 ^= 41%]; see **Figure 3D** for details.

#### LBR

Four studies investigated the LBR after different treatments in the two groups, and the fixed-effects model was utilized for comparison. The results revealed that LBR was increased by G-CSF administration[OR=1.93, 95%CI (1.28,2.92), I^2 ^= 42%]; see **Figure 3E** for details.

### Subgroup analysis

**Figure 4:**
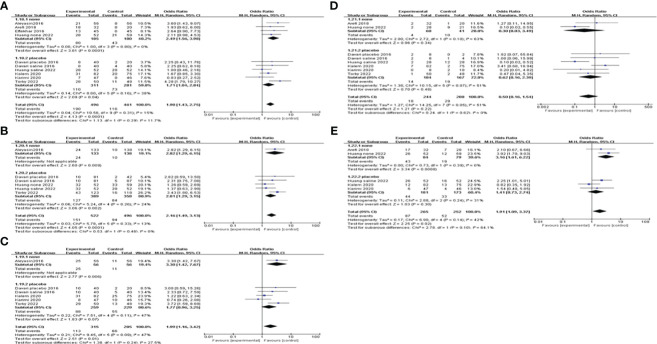
Subgroup analysis comparing the effect of G-CSF on pregnancy outcomes based on different controls. **(A)** clinical pregnancy rate. **(B)** implantation rate. **(C)** biochemical pregnancy rate. **(D)** miscarriage rate. **(E)** live birth rate.

We conducted a subgroup analysis based on different controls. Four studies were blank controls and five were placebo controls (saline or empty catheter entry into the uterine cavity). The results indicated that G-CSF treatment could exert good advantages in improving CPR [OR=2.49, 95%CI (1.56, 3.98), I^2 ^= 0%], IR [OR=2.82, 95%CI (1.29, 6.15)], BPR[OR=3.30, 95%CI (1.42, 7.67)] and LBR [OR=3.16, 95%CI (1.61, 6.22), I^2 ^= 0%] compared with the blank control group. However, compared with placebo controls, G-CSF showed beneficial effects on CPR [OR=1.71, 95%CI (1.04, 2.84), I^2 ^= 38%] and IR [OR=2.01, 95%CI (1.29, 3.15), I^2 ^= 24%], but not on LBR; see **Figure 4** for details.

**Figure 5:**
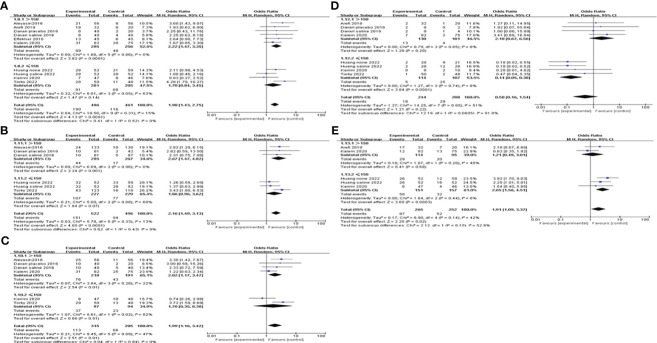
Subgroup analysis comparing the effect of G-CSF on pregnancy outcomes based on different administration dosages. **(A)** clinical pregnancy rate. **(B)** implantation rate. **(C)** biochemical pregnancy rate. **(D)** miscarriage rate. **(E)** live birth rate.

We also did subgroup analysis by administering a dosage of G-CSF on all of the pregnancy outcomes. The pooling results indicated that >150μg of G-CSF treatment could exert good advantages in improving CPR [OR=2.22, 95%CI (1.47, 3.35), I^2 ^= 0%], but ≤150μg could not. Furthermore, both IR and BPR were higher in the >150μg subgroup[IR: OR=2.67, 95%CI (1.47, 4.82), I^2 ^= 0%; BPR: OR=2.02, 95%CI (1.17, 3.47), I^2 ^= 22%], but not in the ≤150μg subgroup. However, in the ≤150μg subgroup, MR was lower [OR=0.14, 95%CI (0.05, 0.38), I^2 ^= 0%], and LBR was improved [OR=2.65, 95%CI (1.56, 4.51), I^2 ^= 0%], but neither of them showed a significant difference in the >150μg subgroup; see **Figure 5** for details.

**Figure 6:**
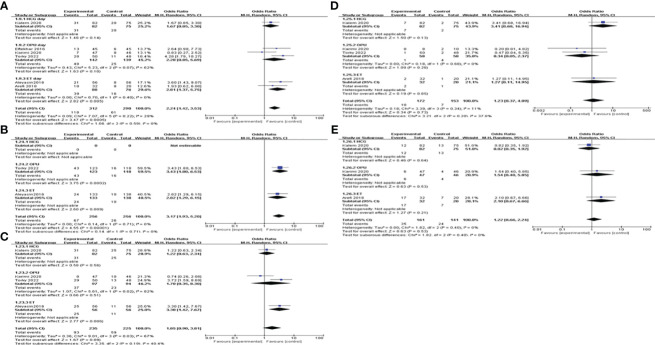
Subgroup analysis comparing the effect of G-CSF on pregnancy outcomes based on different intervention timing. **(A)** clinical pregnancy rate. **(B)** implantation rate. **(C)** biochemical pregnancy rate. **(D)** miscarriage rate. **(E)** live birth rate.

When subgroup analysis was carried out according to different intervention timing, the results showed that G-CSF administration on the day of ET could increase CPR [OR=2.81, 95%CI (1.37, 5.75), I^2 ^= 0%], IR[OR=2.82, 95%CI (1.29, 6.15)]and BPR[OR=3.30, 95%CI (1.42, 7.67)], while not on the day of ovum pick-up (OPU) or HCG injection. MR and LBR of different administration timing subgroups did not differ significantly; see **Figure 6** for details.

**Figure 7:**
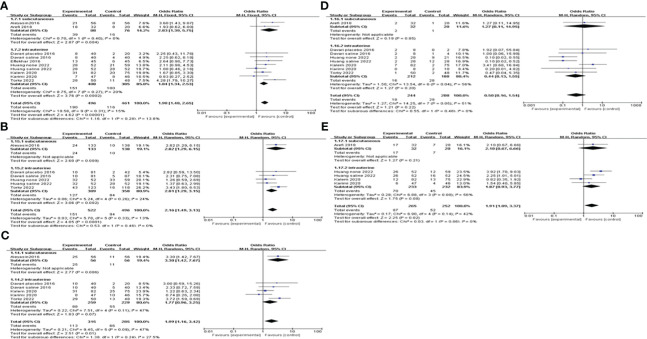
Subgroup analysis comparing the effect of G-CSF on pregnancy outcomes based on the route of G-CSF administration. **(A)** clinical pregnancy rate. **(B)** implantation rate. **(C)** biochemical pregnancy rate. **(D)** miscarriage rate. **(E)** live birth rate.

We conducted a subgroup analysis based on the route of G-CSF administration. The pooling results indicated that both subcutaneous injection and intrauterine infusion of G-CSF improve CPR [subcutaneous OR=2.83, 95%CI (1.39, 5.75), I^2 ^= 0%; intrauterine OR=1.84, 95%CI (1.34, 2.53), I^2 ^= 20%]; and IR [subcutaneous OR=2.82, 95%CI (1.29, 6.15); intrauterine OR=2.01, 95%CI (1.29, 3.15), I^2 ^= 24%]; see **Figure 7** for details.

### G-CSF versus HCG

**Figure 8:**
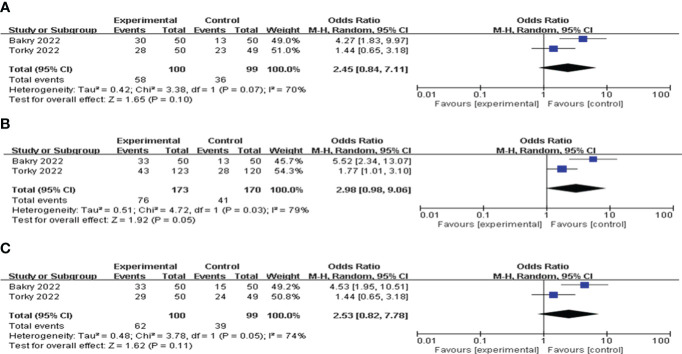
Forest plot comparing the effect of G-CSF on pregnancy outcomes versus HCG. **(A)** clinical pregnancy rate. **(B)** implantation rate. **(C)** biochemical pregnancy rate.

Two articles compared the values of G-CSF with HCG on pregnancy outcomes in patients with a history of implantation failure. Pooled analysis indicated that the CPR, IR and BPR in the G-CSF group were higher than those in the HCG group, but the difference was not statistically significant; see **Figure 8** for details.

### Publication bias analysis

For publication bias, the funnel plot and regression analyses of Egger’s test indicated a relatively low likelihood of publication bias. The result is presented in **Supplementary Figure 1**.

## Discussion

We performed a systematic review and meta-analysis to summarize the efficacy of G-CSF in women who experienced at least one implantation failure. Nine RCTs reported sufficient quantitative data which allowed for statistical pooling. The meta-analysis outcome showed that patients with a history of implantation failure could benefit from the use of G-CSF. When compared to the control group, the use of G-CSF was related to a statistically significant increase in the rates of clinical pregnancy, implantation, chemical pregnancy and live birth. However, G-CSF did not show significant advantages in improving MR. We estimated that such results may be attributed to the single injection of G-CSF, with the decrease in the sustaining effects of G-CSF, some patients with poor endometrial receptivity are unable to maintain pregnancy, leading to the occurrence of miscarriage.

Some studies found that an endometrial thickness of <7 mm and a high reproductive age may negatively impact the pregnancy results (39–42). A meta-analysis to explore the efficiency of G-CSF on infertile women with thin endometrium reported that intrauterine perfusion of G-CSF could significantly improve endometrial thickness and CPR (43). In this review, all of the women included were below 40 years old and had endometrial thicknesses greater than 7mm, so we excluded the influence of endometrium thickness and childbearing age.

Over the last two decades, endometrial injury has been studied to improve implantation rates and decrease the incidence of implantation failure in IVF cycles. A previous meta-analysis has proved that endometrial injury could exert good advantages in improving implantation success, CPR and LBR in patients with at least one failed IVF cycle (44). During the intrauterine infusion G-CSF, the insertion and removal of tubes are needed to finish the treatments. If the controls only took measures of no treatment, it was unable to assess the effect of the mechanical manipulation of the uterine cavity on the pregnancy outcomes. Since some included studies in this meta-analysis were blank controls and some were placebo controls, we conducted a subgroup analysis based on different controls. The results indicated that G-CSF treatment could exert good advantages in improving CPR, IR, and LBR compared with the blank control group. However, compared with placebo controls (saline or empty catheter entry into the uterine cavity), G-CSF showed beneficial effects on CPR and IR, but not on LBR. This suggested that G-CSF therapy had a beneficial influence on CPR and IR, rather than due to mechanical manipulation.

The reasons for this improvement may include induction of local immune regulation of the endometrium, embryo adhesion and implantation, proliferation of trophoblasts and endometrial vascular remodeling by G-CSF. Some studies have reported that the receptor for G-CSF can be found in trophoblastic cells, endometrial glandular cells, follicular cells and oocytes (19–21). Moreover, as an important medium of intercellular communication, G-CSF could recruit dendritic cells, activate Tregs and promote the secretion of Th2 cytokine, which influence the gene expression regulating cellular adhesion pathways, vascular remodeling and immune modulation in the endometrium (45). Recently, Ding JL et al. demonstrated that G-CSF derived from M2 macrophage could promote trophoblasts invasion and migration through activating PI3K/AKT/Erk1/2 pathway, thereby involving in normal pregnancy program (46).

We performed a subgroup analysis by the route of G-CSF administration, and our results showed that both subcutaneous injection and intrauterine infusion of G-CSF improve CPR in women with implantation failure. Consistent with this study, a previous meta-analysis by Hou, et al. reported that the administration of G-CSF via either subcutaneous injection or intrauterine infusion for RIF patients can improve CPR (30). However, it failed to explore the optimal administration dosage of G-CSF. We conducted subgroup analysis by administration dosage of G-CSF, the results suggested that >150μg of G-CSF therapy could exert good advantages in improving CPR, IR, and BPR, but not abortion and LBR. It is interesting that in the ≤150μg subgroup, both MR and LBR were improved, while CPR, IR, and BPR were not significantly different. This is the only study to investigate the relationship between G-CSF dose and pregnancy outcome so far. However, due to the small sample size and indirect evidence, no accurate conclusions can be drawn, and further studies with rigorously designed RCT are needed.

In addition, we noted that the timing of G-CSF administration was inconsistent across the included studies, so we conducted a subgroup analysis according to different intervention timing. The results suggested that G-CSF administration on the day of ET could increase CPR, while not on the day of OPU or HCG injection. This meta-analysis may provide a favorable time point to initiate G-CSF.

On the other hand, when compared to HCG, G-CSF increased the CPR, IR and BPR in patients with implantation failure, but did not achieve a statistical difference. Considerable heterogeneity was observed in the synthesis, which might have been caused by the small number of included studies and small sample size.

## Limitations

There are several limitations of this systematic review and meta-analysis. First, in most of the included studies, the cause of implantation failure was not mentioned, so we were not able to perform a sub-group analysis regarding the cause of implantation failure, and still more studies are needed for a definitive conclusion. Second, not all of the included studies gave the research outcomes that we needed, more well-designed clinical studies are needed to investigate the optimal dosage and timing of G-CSF therapy to improve LBRs. Third, most of the studies we included analyzed fresh cycles, while one compared frozen cycles, and another one included both fresh and frozen cycles. However, we could not extract them from the statistics to conduct a subgroup analysis and draw a conclusion as to which embryo transfer protocol benefits more from G-CSF. At the same time, our included studies did not report whether euploid tests were carried out or not. We suggest that the following studies list more detailed data if they include more than one embryo transfer method. Despite this, our study provides a comprehensive review of the current literature guided by a prospectively registered protocol. Overall, the conclusions drawn from this review represent a current collation of evidence.

## Conclusions

In conclusion, G-CSF has a beneficial effect on CPR to some extent, and the administration dosage and timing influence the effect size. Our findings from indirect evidence support the use of >150μg G-CSF administration on the day of ET in women who experienced at least one implantation failure. Further, head-to-head RCTs with high quality and larger sample sizes are needed to evaluate the effects of different dosages and timing of G-CSF administration on pregnancy outcomes to provide direct evidence.

## Data availability statement

The original contributions presented in the study are included in the article/**Supplementary Material**. Further inquiries can be directed to the corresponding author.

## Author contributions

QS: Writing – review & editing, Writing – original draft, Supervision. ZP: Writing – original draft, Writing – review & editing. RY: Writing – original draft. XL: Writing – original draft.
