# Supplementary figures and images for: The value of G-CSF in women experienced at least one implantation failure: a systematic review and meta-analysis
Source: Front Endocrinol (Lausanne). 2024 Apr 16;15:1370114. doi: 10.3389/fendo.2024.1370114 (PMC11061619; doi:10.3389/fendo.2024.1370114)

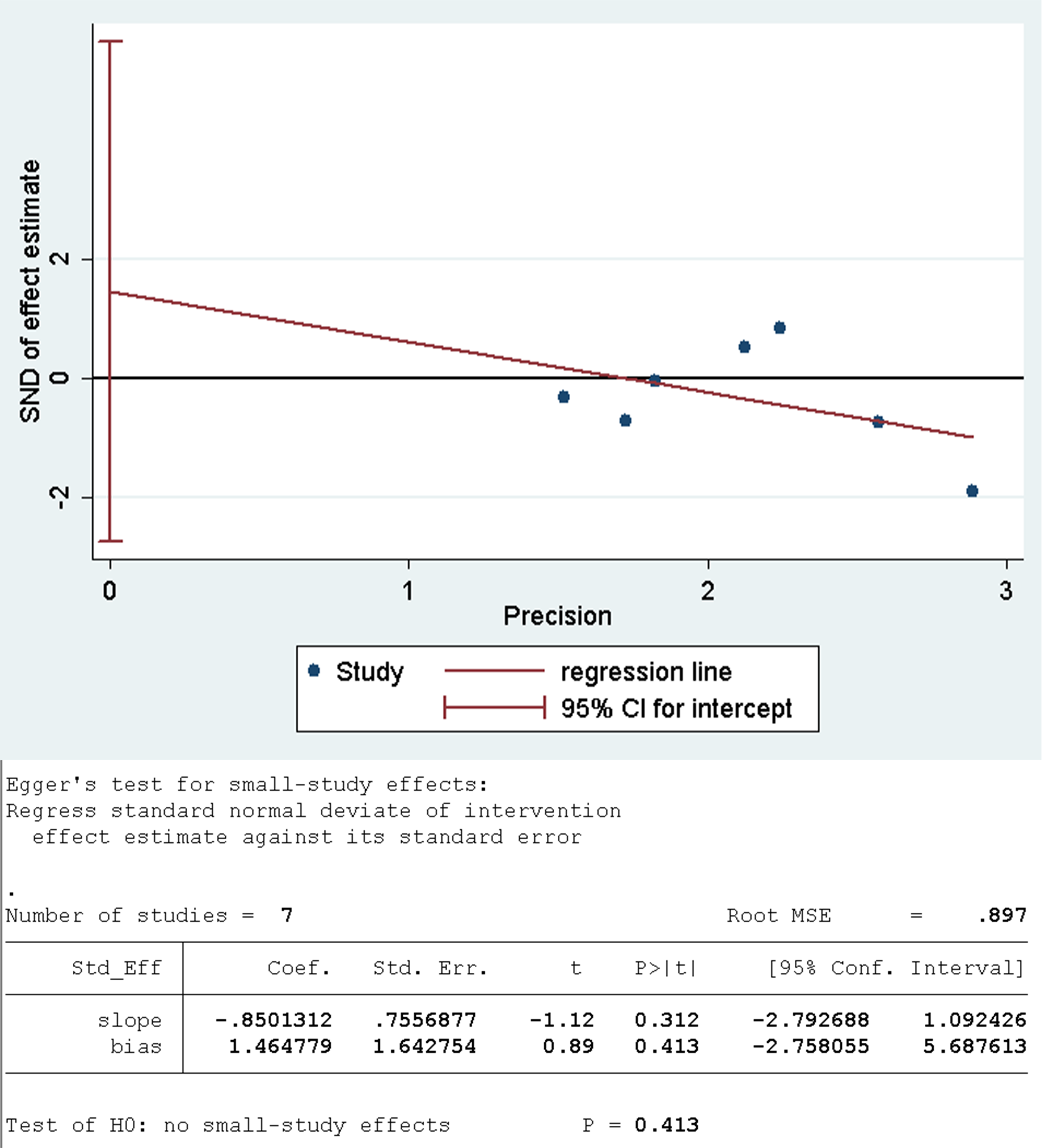

Supplement: Supplementary file 1 [file Image_1.tif]
